# The Role of Artificial Intelligence in Prediction, Risk Stratification, and Personalized Treatment Planning for Congenital Heart Diseases

**DOI:** 10.7759/cureus.44374

**Published:** 2023-08-30

**Authors:** Syed Naveed Mohsin, Abubakar Gapizov, Chukwuyem Ekhator, Noor U Ain, Saeed Ahmad, Mavra Khan, Chad Barker, Muqaddas Hussain, Jahnavi Malineni, Afif Ramadhan, Raghu Halappa Nagaraj

**Affiliations:** 1 General Surgery, Cavan General Hospital, Cavan, IRL; 2 General Surgery, American University of Antigua, Brooklyn, USA; 3 Neuro-Oncology, New York Institute of Technology, College of Osteopathic Medicine, Old Westbury, USA; 4 Medicine, Mayo Hospital, Lahore, PAK; 5 Medicine, King Edward Medical University, Lahore, PAK; 6 Medicine and Surgery, Mayo Hospital, Lahore , PAK; 7 Public Health, University of South Florida, Tampa, USA; 8 Internal Medicine, Mayo Hospital, Lahore, PAK; 9 Medicine and Surgery, Maharajah's Institute of Medical Sciences, Vizianagaram, IND; 10 Medicine, Universal Scientific Education and Research Network (USERN), Yogyakarta, IDN; 11 Medicine, Faculty of Medicine, Public Health, and Nursing, Gadjah Mada University, Yogyakarta, IDN; 12 Surgery, Avalon University School of Medicine, Willemstad, CUW

**Keywords:** personalized treatment, deep learning, cardiology, artificial intelligence, congenital heart diseases

## Abstract

This narrative review delves into the potential of artificial intelligence (AI) in predicting, stratifying risk, and personalizing treatment planning for congenital heart disease (CHD). CHD is a complex condition that affects individuals across various age groups. The review highlights the challenges in predicting risks, planning treatments, and prognosticating long-term outcomes due to CHD's multifaceted nature, limited data, ethical concerns, and individual variabilities. AI, with its ability to analyze extensive data sets, presents a promising solution. The review emphasizes the need for larger, diverse datasets, the integration of various data sources, and the analysis of longitudinal data. Prospective validation in real-world clinical settings, interpretability, and the importance of human clinical expertise are also underscored. The ethical considerations surrounding privacy, consent, bias, monitoring, and human oversight are examined. AI's implications include improved patient outcomes, cost-effectiveness, and real-time decision support. The review aims to provide a comprehensive understanding of AI's potential for revolutionizing CHD management and highlights the significance of collaboration and transparency to address challenges and limitations.

## Introduction and background

Congenital heart disease (CHD) is a complex condition affecting various age groups, with up to 4 to 5/1,000 live births affected by its diverse structural abnormalities [1]. Overcoming challenges like heterogeneity, limited data, ethical considerations, and individual variability in CHD management requires a multidisciplinary approach and innovative strategies. In this context, artificial intelligence (AI) has gained prominence as a transformative technology that can revolutionize the diagnosis, prognosis, and treatment of CHD [2].

The capacity of AI to analyze extensive datasets, recognize patterns, and generate insights has immense implications for CHD management. It excels in predicting CHD occurrence and progression, enabling early interventions and preventive measures. The technology aids in risk stratification by factoring in patient-specific variables, facilitating personalized monitoring, and targeting interventions [3,4]. AI optimizes diagnosis, treatment planning, and outcomes, ushering in a new era of CHD management [5]. The purpose of this narrative review is to comprehensively examine the role of AI in predicting CHD, understanding risk factors, and facilitating personalized treatment planning. By consolidating and summarizing existing research, the review aims to elucidate the potential benefits and challenges of integrating AI into CHD management. It also explores how AI algorithms analyze diverse datasets to predict and understand CHD risk factors. Additionally, the review investigates how AI contributes to risk stratification by considering patient-specific factors associated with CHD complications.

Furthermore, this narrative review delves into the application of AI in crafting personalized treatment strategies for CHD patients. By assessing individual patient traits, therapeutic responses, and treatment outcomes, AI aids in formulating tailored and evidence-based plans. It also discusses the potential of AI in optimizing surgical procedures, drug selection, and exploring novel therapies [6]. Ultimately, this article aims to offer a comprehensive overview of the latest advancements and challenges related to the integration of AI into CHD management. By presenting synthesized research findings, the review aims to inform healthcare professionals, researchers, and policymakers about the potential impact of AI on enhancing CHD outcomes. Through its insights, this review seeks to advance our understanding of the role of AI in improving the lives of individuals affected by congenital heart diseases.

## Review

Prediction of CHDs 

AI is increasingly proving transformative across various sectors, including healthcare, particularly in the realm of diagnosing and managing CHDs. The potential applications of AI encompass a range of critical functions in the early detection and prognosis of CHD. These tasks include image acquisition, optimization, automated measurements, outlier detection, diagnostic classification, and outcome prediction [7]. The contributions of AI address the limitations of traditional 2D fetal imaging techniques, which often fall short of providing conclusive information due to fetal movement, heart size, and the expertise of the sonographer [8].

The integration of AI and machine learning (ML) has significantly improved the visualization of cardiac defects. By processing data and identifying patterns, AI algorithms can enhance the accuracy of fetal heart pictures and assist in evaluating cardiac form and function. This is particularly relevant given the challenges posed by factors like rapid fetal heartbeat, small heart size, and limited access to the fetus. The applications of AI in pediatric cardiology are multifaceted and extend to clinical examination, image processing, fetal cardiology, risk assessment, precision cardiology, and treatment planning. Machine learning algorithms have shown promise in diagnosing both critical and non-critical CHDs [9]. In recent years, various AI models have gained traction, yielding promising breakthroughs.

One notable achievement is the combination of human-performed ultrasound (US) and machine analysis, leading to a remarkable 95% detection rate of CHDs in a test sample [10]. This suggests the potential of automated prenatal screening to significantly reduce newborn mortality by identifying CHDs early. Moreover, AI-enabled algorithms can estimate prognosis for conditions like tetralogy of Fallot, enhancing the accuracy of mortality risk assessments and making the process more efficient [11]. The utility of AI is not limited to diagnosis; it extends to training and education. AI-powered software can offer a second view to sonographers during fetal echocardiography, providing valuable guidance in the absence of experienced colleagues. This technology can be integrated into ultrasound machines, allowing real-time assistance during examinations [12].

AI algorithms come in various forms, including deep learning (DL), a subset of ML that employs artificial neural networks for complex tasks such as object recognition and speech analysis [13]. The application of DL spans sectors like healthcare, where it aids in tasks ranging from diagnostic prediction to outcome forecasting. Prediction models are central to healthcare research and practice. In cardiology, ML-based prediction models are gaining traction for forecasting outcomes and supporting clinical decisions [14,15]. For instance, these models have been employed to predict mortality following heart transplantation in adults with congenital heart disease, aiding physicians in personalized treatment strategies [15]. While AI holds immense potential in healthcare, including pediatric cardiology, there is still a need for further research to develop models that are interpretable, reliable, and applicable to a diverse range of complex CHD cases. The rapid growth of AI in clinical settings enhances doctors' capabilities, standardizes care, and drives advancements in patient outcomes.

Risk stratification of CHDs

The potential benefits of AI extend to various domains of healthcare, including adult CHD. Developing and using risk scores based on AI predictions can enhance patient care by guiding appropriate follow-up frequencies, treatment escalations, and referrals for procedures or transplantation [16]. The impact of AI on auscultatory results can improve diagnosis accuracy, while ensemble learning models can aid in diagnosing fetal congenital heart defects through imaging [7].

In the field of pediatric cardiology, AI-based algorithms play a vital role in clinical assessment, diagnosis, procedure planning, and intervention management [9]. These algorithms also facilitate data extraction from wearables for ambulatory health monitoring and risk assessment. Machine learning models demonstrate the ability to predict various outcomes, such as hospital length of stay, ventilator support time, and mortality after congenital heart surgery. Interpretable machine learning approaches enable accurate predictions of post-surgery complications and patient-specific factors contributing to these predictions. By combining interpretability and model performance, AI empowers clinicians to make more informed decisions. For neonates who undergo complex procedures, AI-driven risk prediction models aid in forecasting one-year death or cardiac transplantation risks, as well as extended hospital stay risks [17,18].

Several AI models, including Multilayer Perceptron, Random Forest, Extra Trees, Stochastic Gradient Boosting, Ada Boost Classification, and Bag Decision Trees, have demonstrated success in the medical literature [19,20]. These models are employed for risk prediction, leveraging factors such as diagnostic groups, patient attributes, and medical history to achieve high levels of accuracy.

Personalized treatment for CHDs

AI is transforming the field of CHD by enabling precision medicine through the analysis of large datasets from these patients. This approach holds the potential to revolutionize diagnosis, treatment planning, and interventions for individual patients. The ability of AI to amalgamate diverse layers of medical data, including clinical records, imaging data, environmental factors, and social influences, offers personalized analytics that can improve patient outcomes. Precision medicine AI in precision medicine for CHD is revolutionizing diagnosis and treatment by leveraging vast datasets to provide personalized care. The integration of AI into CHD management is reshaping the landscape of healthcare, offering the potential for more accurate diagnoses, tailored treatment strategies, and improved patient outcomes.

At the core of AI's impact is its ability to process large amounts of data from CHD patients, including clinical information, environmental factors, imaging data, and social influences. This wealth of data serves as the foundation for developing predictive models that can guide individualized treatment plans. For instance, serum metabolite panels and DNA methylation are being explored as tools for CHD screening, showcasing the potential of AI in identifying unique biomarkers for early detection [21,22]. Digital twin technology takes personalization a step further by creating virtual patient models that mirror real-time clinical data. By combining mechanistic and statistical models, these virtual counterparts allow clinicians to make more informed decisions and predictions. This technology integrates data from mobile health monitoring, clinical reports, and medical imaging to enhance clinical insights and offer tailored interventions [23].

The potential of AI is evident in studies that utilize deep learning algorithms to diagnose, predict prognosis, and direct therapy for adult patients with CHD. With impressive accuracy rates, these algorithms analyze vast medical records to provide insights into diagnosis, disease complexity, and disease progression [24]. Diller et al. investigated how deep learning-based algorithms can be used to diagnose, predict prognosis, and direct therapy in adult patients with congenital cardiac disorders. Over the course of 18 years, the deep learning-based system was applied to over 44,000 medical records from over 10,000 individuals. The analysis was utilized to describe the diagnosis, disease complexity, and New York Heart Association (NYHA) class of adult congenital heart disease, and it demonstrated accuracy of 91.1%, 97.0%, and 90.6%, respectively, in the test sample [6]. This exemplifies the role of AI in refining patient care and treatment strategies.

AI techniques are employed in multiple facets of personalized treatment planning for CHD patients. AI techniques like reinforcement learning, supervised learning, and unsupervised learning are used to analyze extensive datasets, uncover patterns, and generate predictive models [25]. These models enhance diagnostic accuracy, early disease detection, and specialized cardiac care access, offering valuable insights for neonatal and pediatric CHD populations. AI algorithms analyze medical imaging data, such as cardiac MRIs and echocardiograms, to precisely identify anatomical changes and abnormalities. Techniques like picture segmentation and feature extraction contribute to personalized treatment regimens based on individual anatomical traits [26]. Natural language processing (NLP) methods extract valuable information from unstructured data sources like electronic health records and clinical notes. This information aids in creating individualized treatment plans and provides a comprehensive understanding of patients' conditions, facilitating better clinical decisions [27].

AI algorithms utilize patient-specific variables to develop risk prediction models. These models calculate probabilities of complications, disease progression, and treatment outcomes, guiding individualized treatment plans based on unique risk profiles [28]. AI-powered decision support systems offer clinicians recommendations for treatments based on patient-specific data and available research. These systems optimize resource allocation, minimize errors, and enhance patient outcomes by providing evidence-based suggestions [29]. Reinforcement learning techniques tailor treatment plans through trial and error, modeling the patient's state and treatment choices. This adaptive approach responds to patient feedback and preferences. Collaborative filtering techniques consider patient preferences by analyzing data from similar patients, enabling the customization of treatment regimens based on individual preferences and histories [30].

AI-driven decision support systems offer several advantages for CHD treatment. The ability of AI to analyze extensive patient data enhances the accuracy and precision of diagnosis and treatment recommendations. AI algorithms consider various factors to provide personalized treatment strategies that consider anatomical variations, comorbidities, and patient preferences. Decision support systems rely on evidence-based medicine and the latest clinical recommendations, ensuring patients receive the most current and effective treatments [31]. These systems optimize resource utilization, leading to efficient allocation of healthcare resources and cost savings. AI systems provide alerts and reminders for potential risks, reducing the likelihood of medical errors.

Ethical considerations and challenges

The utilization of AI in the prediction, risk stratification, and personalized treatment planning for CHD introduces a range of ethical considerations. These considerations encompass aspects like privacy, informed consent, bias, ongoing monitoring, and the role of human judgment. The application of AI, particularly in cardiovascular medicine, raises concerns about patient data privacy and security [32]. Protecting sensitive patient information is crucial when using AI for CHD therapy planning. Adhering to data privacy laws and ensuring secure data sharing are vital for maintaining patient trust and safeguarding their confidential information. Patients with CHD should be well informed about the involvement of AI in their treatment planning. Transparent communication is essential in explaining how AI algorithms analyze their data, the purpose of the analysis, potential benefits, and associated risks [33]. Patients have the right to provide informed consent for their data to be used in AI-driven treatment plans.

Algorithmic bias is a significant concern, as AI systems trained on biased datasets can lead to biased predictions and recommendations [34]. Ensuring that AI algorithms are regularly monitored and audited for both data and algorithmic bias is crucial to preventing unfair and inaccurate results that could disproportionately affect certain groups. Ongoing monitoring and evaluation of AI systems are necessary to ensure their effectiveness, accuracy, and safety. Regular assessments should include checks for fairness, robustness, and clinical relevance. Transparent reporting of system performance and prompt updates to address any identified issues are essential to maintaining the credibility of AI-driven treatment planning [35]. AI should complement, not replace, healthcare professionals. Human oversight and clinical judgment remain essential in the treatment planning process. AI should be viewed as a supportive tool that aids clinicians in making informed decisions, rather than a substitute for their expertise. Ultimately, clinicians should retain the authority to make treatment decisions based on their clinical knowledge and patient preferences [36].

Effective AI model training relies on comprehensive and diverse datasets. However, data availability can be limited, particularly for rare CHD conditions. Insufficient data can hinder the development of accurate AI algorithms and affect their performance and applicability [28]. Some AI algorithms, especially deep learning models, may lack interpretability, making it challenging for clinicians to understand the rationale behind treatment recommendations [37]. Bridging the gap in understanding between clinicians and AI models is crucial to fostering trust and adoption. AI models developed in one context may not generalize well to different patient populations. Variations in demographics and healthcare practices can impact the performance of the model. Extensive validation across diverse populations is essential to ensuring the broad applicability of AI algorithms [38]. Transitioning from research settings to real-world clinical practice poses challenges for AI implementation. The complexities of real-world clinical scenarios may not be fully captured in controlled research environments, necessitating thorough validation in actual healthcare settings [39].

Addressing these challenges requires collaborative efforts among researchers, healthcare professionals, policymakers, and regulatory authorities. To fully harness the potential of AI in CHD treatment planning, the development of interpretable AI algorithms, the improvement of data quality and diversity, and comprehensive real-world validations are essential steps forward. By navigating these ethical considerations and limitations, AI has the potential to revolutionize the management of congenital heart diseases and enhance patient outcomes.

Future directions and implications

The application of AI in the realm of CHDs holds great potential for enhancing prediction, risk assessment, and personalized treatment planning. However, several key challenges must be addressed to fully leverage AI's capabilities and ensure its seamless integration into clinical practice.

One notable challenge is the need for larger and more diverse datasets [28]. The effectiveness of AI models heavily relies on the quality and quantity of data they are trained on. Current datasets related to CHD often lack diversity and comprehensiveness, hindering the development of accurate and generalizable AI algorithms. Collaborative efforts should be prioritized to establish multi-center partnerships and data-sharing initiatives. This approach can lead to more robust machine learning (ML) models capable of providing more accurate predictions and insights [40]. Diversifying the data sources used for AI modeling is another crucial avenue for future research. While imaging data like echocardiograms are commonly used, other data streams, such as genetic information, clinical variables, and patient-reported outcomes, remain largely untapped. The integration of these disparate data sources through interoperable platforms can lead to comprehensive patient profiles, enabling more accurate predictive models and treatment plans [41-43].

A critical aspect that demands attention is the dynamic nature of CHD and the need for longitudinal analysis. CHD patients undergo various interventions, and their condition evolves. AI models should be designed to analyze longitudinal data, offering insights into disease progression, treatment effectiveness, and long-term outcomes. By adapting to these changes and continuously learning from new data, AI can provide real-time predictions and recommendations, aiding clinicians in making informed decisions [44]. Prospective validation of AI models within real-world clinical settings is imperative to ensure their reliability and effectiveness. Large-scale clinical trials are necessary to assess the impact of AI-driven decision support systems on patient outcomes and healthcare resource utilization. Transparency and interpretability are paramount when implementing AI in clinical decision-making. Developing explainable AI algorithms can foster trust among clinicians by providing insights into the rationale behind model predictions [28].

The integration of AI into clinical practice has profound implications. First, it can lead to improved patient outcomes by enabling early intervention, risk stratification, and tailored treatment planning. This, in turn, can reduce complications and enhance the quality of life for CHD patients [43]. Additionally, AI can enhance cost-effectiveness in healthcare by optimizing resource allocation and preventing unnecessary procedures. The ability to generate personalized treatment plans based on individual characteristics can minimize adverse effects and improve intervention efficacy. Real-time decision support through AI can empower clinicians to make well-informed choices, further enhancing patient care [45].

In conclusion, the use of AI in CHD offers transformative possibilities for patient care, but overcoming challenges related to data, dynamic modeling, and validation is crucial. By embracing collaborations, diversifying data sources, and ensuring transparency, the full potential of AI can be harnessed, resulting in improved patient outcomes, efficient resource allocation, and personalized treatment approaches.

## Conclusions

In the rapidly evolving landscape of healthcare, the convergence of AI and CHD management holds great promise. This comprehensive narrative review underscores the potential of AI to transform prediction, risk stratification, and personalized treatment planning for CHD. The intricate nature of CHD, coupled with limited data and ethical considerations, necessitates innovative solutions. The ability of AI to analyze vast datasets, identify patterns, and generate insights offers a path forward. The review emphasizes the critical importance of collaboration, diverse datasets, and longitudinal analysis to overcome challenges and unlock AI's full potential. While AI offers groundbreaking opportunities, ethical considerations surrounding privacy, bias, transparency, and the role of human judgment cannot be overlooked. This intersection of AI and CHD management demands a delicate balance, where AI serves as a tool to complement clinical expertise. As the healthcare landscape continues to evolve, AI's role in CHD management will undoubtedly expand. By navigating challenges, harnessing collaborative efforts, and ensuring ethical adherence, the consolidation of AI and CHD management holds the promise to revolutionize care, enhance patient outcomes, and create a future where precision medicine becomes a reality for all individuals affected by congenital heart diseases.
